# Supplementary material for: Lineage-specific microbial protein prediction enables large-scale exploration of protein ecology within the human gut
Source: Nat Commun. 2025 Apr 3;16:3204. doi: 10.1038/s41467-025-58442-w (PMC11968815; doi:10.1038/s41467-025-58442-w)
Supplement: Supplementary file 1 — Supplementary Information [file 41467_2025_58442_MOESM1_ESM.pdf]

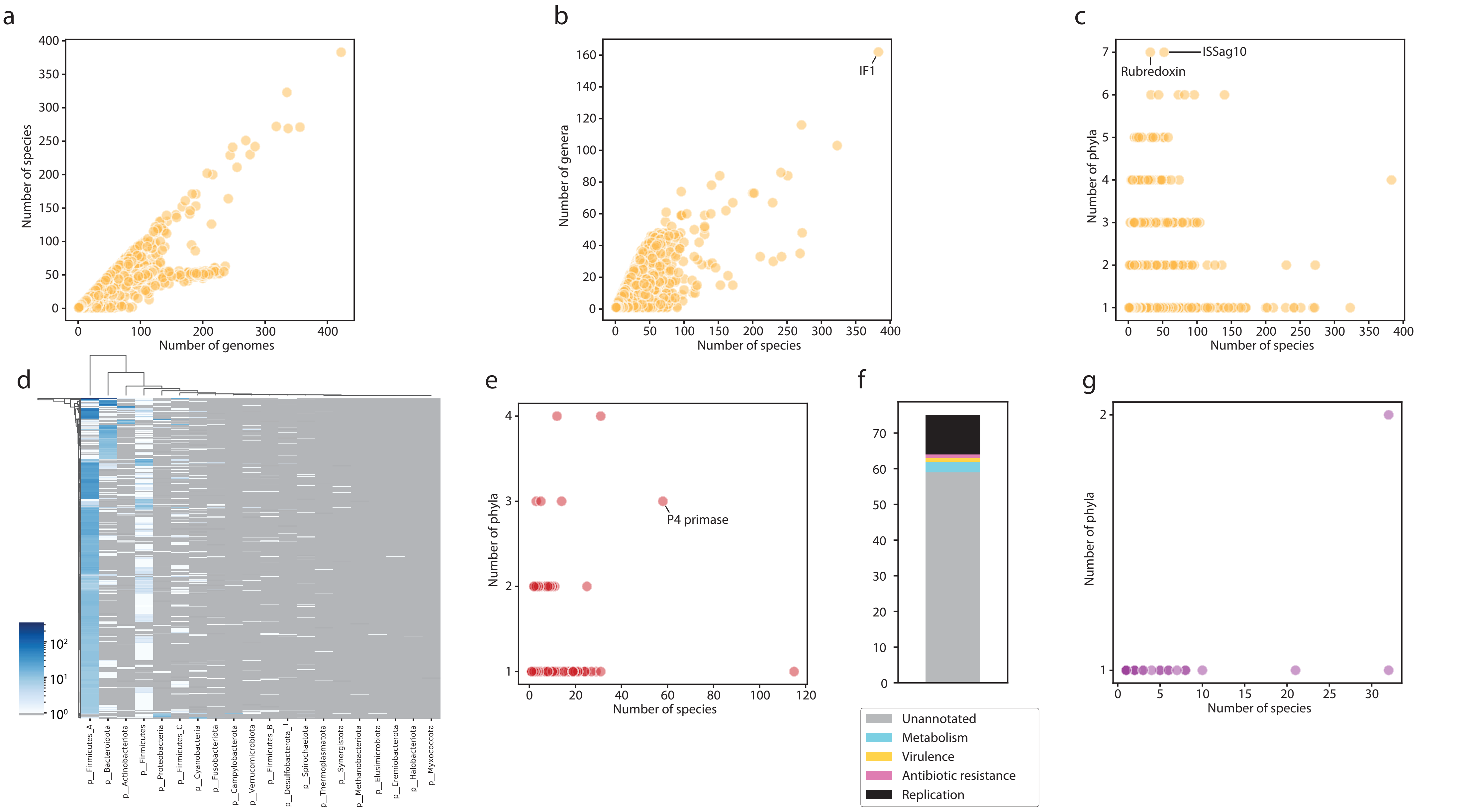

Supplementary Figure 1: Shared functional capacity across taxa within the human gut. **a**: For each protein cluster, their occurrence within unique species and genomes was plotted against each other. **b**: Protein cluster occurrence within unique species and genera was plotted against each other. **c**: Protein cluster occurrence within unique species and phyla was plotted against each other. **d**: The frequency of shared protein clusters between phyla was plotted. Grey represents no shared protein clusters, while darker shades of blue indicate a greater number. **e**: Occurrence of protein clusters containing a sequence of viral origin, within unique species, and phyla. **f**: The functionality of viral containing protein clusters shared between at least 2 phyla (n = 75), based on curated groupings of GhostKOALA assignments, Supplementary Table 7. **g**: Occurrence of protein clusters containing a sequence of eukaryotic origin, within unique species, and phyla.

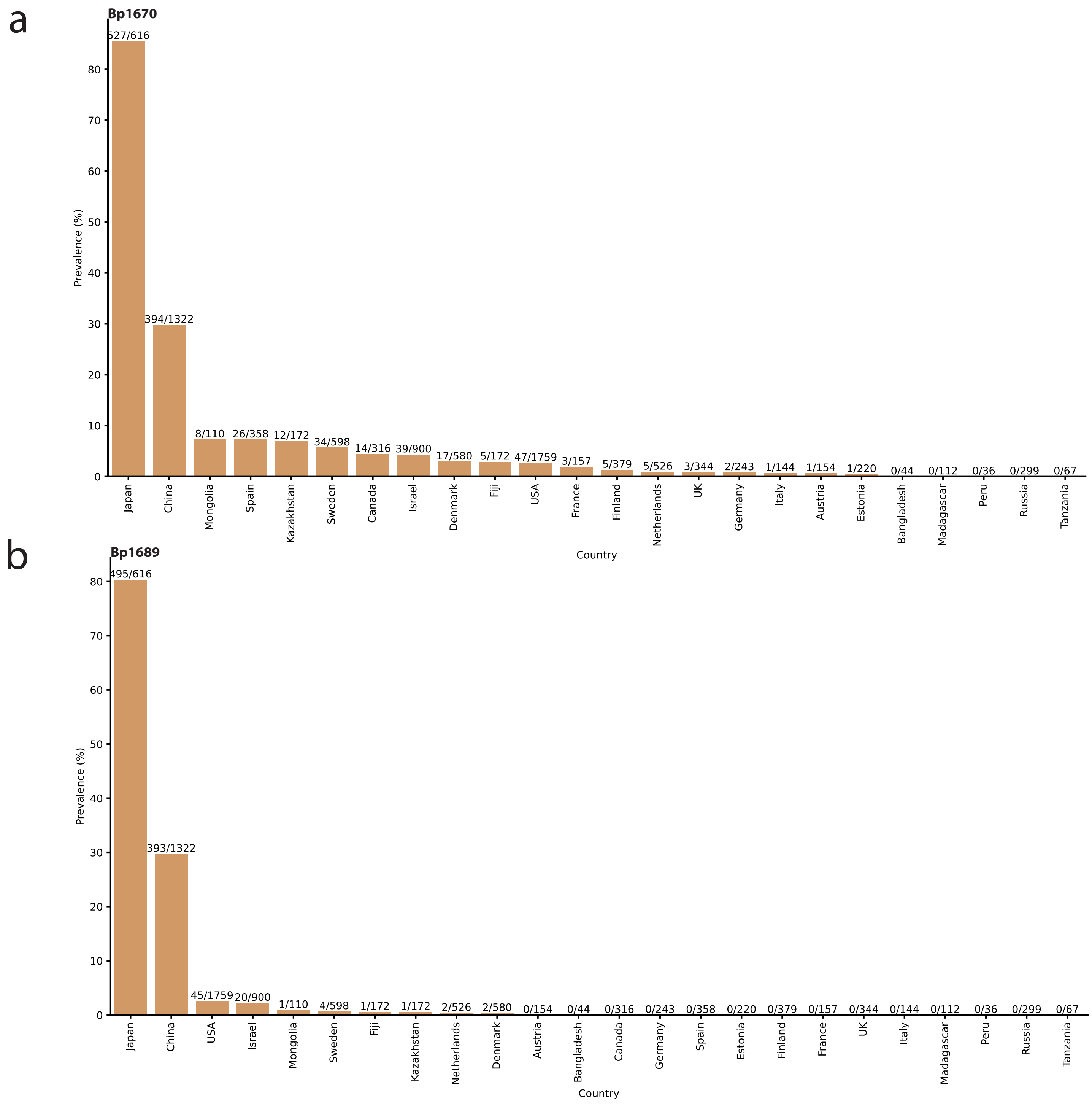

Supplementary Figure 2: Geographical prevalence of seaweed degrading porphyranases (Bp1689) (**a**), and agarases (Bp1670) (**b**). Each protein was studied using InvestiGUT at the default 90% identity threshold.

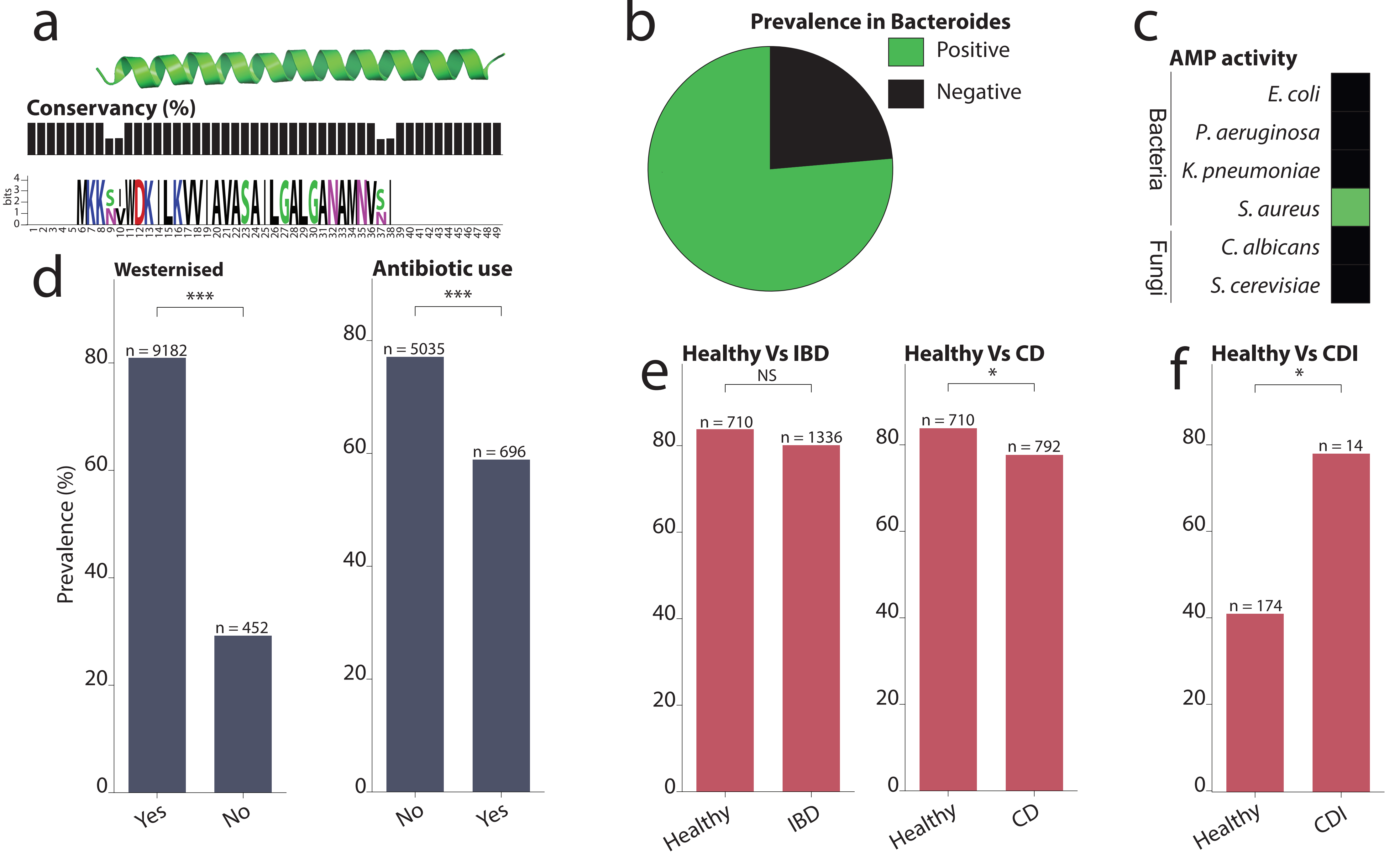

Supplementary Figure 3: Analysis of the small protein cluster '33977|YachidaS_2019__SAMD00114748|Bacteria|11|MetaGeneAnnotator-MetaGeneMark-Pyrodigal'. **a**: The conservation of sequence between members of the cluster, along with an Alphafold2 structural model were generated. **b**: The frequency of the cluster across Bacteroides spp. (n = 59). **c**: The predicted antimicrobial activity against key pathogens and fungi. **d-f**: The prevalence of the cluster with host parameters, including westernised status, and antibiotic use, was studied (**d**), along with the prevalence in IBD and Crohn's disease (CD) patients (**e**), and Clostridioides difficile infection (CDI) patients compared to healthy controls (**f**). Significance from Fisher's exact test, after Benjamini-Hochberg correction, are shown as: NS = not significant, * < 0.05, ** < 0.01, *** < 0.001.
