# Supplementary material for: Lineage-specific microbial protein prediction enables large-scale exploration of protein ecology within the human gut
Source: Nat Commun. 2025 Apr 3;16:3204. doi: 10.1038/s41467-025-58442-w (PMC11968815; doi:10.1038/s41467-025-58442-w)
Supplement: Supplementary file 2 — Description of Additional Supplementary Files [file 41467_2025_58442_MOESM2_ESM.docx]

**Description of Additional Supplementary Files**

**File Name: Supplementary Data 1**

**Description: Gene Prediction Software.** For each gene prediction tool studied, the version, target taxa, and reference are provided.

**File Name: Supplementary Data 2**

**Description: Species studied for optimisation of gene prediction.** For each species studied, the genome name, domain, and database are defined. Those species placed in the ‘probiotic’ and ‘pathogen’ groups are also defined.

**File Name: Supplementary Data 3**

**Description: Comparison of gene prediction tools.** For each genome assigned to the studied domain (Archaeal, Bacterial, Eukaryota, Viral) tools were tested individually, in pairs, and triplets. The perfect, partial, and spurious gene predictions as determined by ORForise are reported.

**File Name: Supplementary Data 4**

**Description: Human gut metagenomic studies analysed.** For metagenomic study used to create MiProGut and the InvestiGUT database, the ‘curated metagenomic data’ identifier, number of included metagenomes (post filtering), and the studies PubMed identifier are provided.

**File Name: Supplementary Data 5**

**Description: Taxonomic assignment of metagenomic contigs.** The number of contigs assigned to Archaea, Bacteria, Eukaryota, Viruses, or defined as unknown are provided, both for each individual sample, and grouped by study.

**File Name: Supplementary Data 6**

**Description: Variation in taxonomic assignment due to Kraken 2 database selection.** The number of contigs assigned to Archaea, Bacteria, Eukaryota, Viruses, or defined as unknown are provided when annotated with either the custom or k2_core_nt_20240904 database.

**File Name: Supplementary Data 7**

**Description: Taxonomic assignment of predicted proteins.** The number of proteins predicted from contigs assigned to each taxonomic group is stated for each metagenomic study.

**File Name: Supplementary Data 8**

**Description: Comparison of protein overlap between MiProGut and UHGP split by domain assignment.** The protein clusters in MiProGut which contain proteins predicted from contigs assigned to each of the domains of life are separated. For each domain, the protein clusters that matched to proteins in UHGP, and those that showed no similarity, are stated.

**File Name: Supplementary Data 9**

**Description: Functional annotation of horizontally transferred proteins.** For all proteins the GHOSTkoala annotations are provided, and for specific functions additional results are provided, including broad-grouping, or further annotation with BLAST comparison against the non-redundant database.

**File Name: Supplementary Data 10**

**Description: Differential expression analysis of frequently expressed small proteins between ulcerative colitis and healthy samples.** For each of the 69 small protein clusters expressed within at least 90% of studied metatranscriptomic samples, the RPKM within ulcerative colitis and healthy samples was calculated and compared. Significantly differentially expressed (adj. P-value < 0.05; Benjamini-Hochberg correction) protein clusters are reported along with their mean RPKM within each group and log2 fold change between groups.

**File Name: Supplementary Data 11**

**Description: Differential expression analysis of frequently expressed small proteins between Crohn’s disease and healthy samples.** For each of the 69 small protein clusters expressed within at least 90% of studied metatranscriptomic samples, the RPKM within Crohn’s disease and healthy samples was calculated and compared. Significantly differentially expressed (adj. P-value < 0.05; Benjamini-Hochberg correction) protein clusters are reported along with their mean RPKM within each group and log2 fold change between groups.
