# Supplementary material for: Lineage-specific microbial protein prediction enables large-scale exploration of protein ecology within the human gut
Source: Nat Commun. 2025 Apr 3;16:3204. doi: 10.1038/s41467-025-58442-w (PMC11968815; doi:10.1038/s41467-025-58442-w)
Supplement: Supplementary file 14 — Reporting Summary [file 41467_2025_58442_MOESM14_ESM.pdf]

nature portfolio

Corresponding author(s): Thomas Hitch

Last updated by author(s): Jan 7, 2025

Reporting Summary

Nature Portfolio wishes to improve the reproducibility of the work that we publish. This form provides structure for consistency and transparency in reporting. For further information on Nature Portfolio policies, see our Editorial Policies and the Editorial Policy Checklist.

Statistics

For all statistical analyses, confirm that the following items are present in the figure legend, table legend, main text, or Methods section.

n/a	Confirmed	
☐	☒	The exact sample size (*n*) for each experimental group/condition, given as a discrete number and unit of measurement
☐	☒	A statement on whether measurements were taken from distinct samples or whether the same sample was measured repeatedly
☐	☒	The statistical test(s) used AND whether they are one- or two-sided<br>*Only common tests should be described solely by name; describe more complex techniques in the Methods section.*
☒	☐	A description of all covariates tested
☐	☒	A description of any assumptions or corrections, such as tests of normality and adjustment for multiple comparisons
☐	☒	A full description of the statistical parameters including central tendency (e.g. means) or other basic estimates (e.g. regression coefficient) AND variation (e.g. standard deviation) or associated estimates of uncertainty (e.g. confidence intervals)
☐	☒	For null hypothesis testing, the test statistic (e.g. *F*, *t*, *r*) with confidence intervals, effect sizes, degrees of freedom and *P* value noted<br>*Give P values as exact values whenever suitable.*
☒	☐	For Bayesian analysis, information on the choice of priors and Markov chain Monte Carlo settings
☒	☐	For hierarchical and complex designs, identification of the appropriate level for tests and full reporting of outcomes
☒	☐	Estimates of effect sizes (e.g. Cohen's *d*, Pearson's *r*), indicating how they were calculated

Our web collection on statistics for biologists contains articles on many of the points above.

Software and code

Policy information about availability of computer code

Data collection	All data used within the analysis was open source and the predicted proteins are provided as a resource. curatedMetagenomicData R package (v3.6.2) used for metadata.
Data analysis	The analysis methods used are detailed within the methods, but a range of open source gene prediction tools were applied to metagenomic assemblies. Using this data, and the associated metadata for the metagenomic samples, we developed InvestiGUT (https://github.com/Matt-Schmitz/InvestiGut), an open source tool for studying protein ecology. This has been made available on GitHub and all resources needed to rerun our analysis are detailed on GitHub as well. Data was analysed using; AUGUSTUS v3.3, GlimmerHMM v3.0.4, SNAP v2006-07-28, Pyrodigal (v2.1.0), ORForise (v1.4.2), BBMap (v38.18) , MEGAHIT (v1.2.9) ,Kraken 2 (v2.1.2) GTDB-Tk (v2.3.2; r214), DIAMOND (v2.1.11) , MMseqs2 (v14-7e284) , eggNOG-mapper (v2.1.12), MANTIS (v1.5.5) , DBAASP toolkit (v3).

For manuscripts utilizing custom algorithms or software that are central to the research but not yet described in published literature, software must be made available to editors and reviewers. We strongly encourage code deposition in a community repository (e.g. GitHub). See the Nature Portfolio guidelines for submitting code & software for further information.

Data

Policy information about availability of data

All manuscripts must include a data availability statement. This statement should provide the following information, where applicable:

- Accession codes, unique identifiers, or web links for publicly available datasets
- A description of any restrictions on data availability
- For clinical datasets or third party data, please ensure that the statement adheres to our policy

Gene and protein predictions, along with the nonredundant MiProGut catalogue at 50%, 90%, 95%, and 100% protein identity, are available at: https://zenodo.org/doi/10.5281/zenodo.10988030. InvestiGUT, along with the protein prediction pipeline used in this work is available at: https://github.com/Matt-Schmitz/InvestiGut.

Data used was from the CuratedMetagenomicData project (https://github.com/waldronlab/curatedMetagenomicDataCuration/tree/master), the assemblies were obtained from Passolli et al (http://segatalab.cibio.unitn.it/data/Pasolli_et_al.html), and the Leviatan et al (2022) representative genomes.

Research involving human participants, their data, or biological material

Policy information about studies with human participants or human data. See also policy information about sex, gender (identity/presentation), and sexual orientation and race, ethnicity and racism.

Reporting on sex and gender	Sex used as metadata information that was included in the open source data we obtained.
Reporting on race, ethnicity, or other socially relevant groupings	Geographical location, but not race or ethnicity was included in the open source metadata, and analysed to understand global trends.
Population characteristics	Geographical location was included in the open source metadata, and analysed to understand global trends.
Recruitment	All data analysed has previously been published and is open source.
Ethics oversight	All data analysed has previously been published and is open source.

Note that full information on the approval of the study protocol must also be provided in the manuscript.

Field-specific reporting

Please select the one below that is the best fit for your research. If you are not sure, read the appropriate sections before making your selection.

☒ Life sciences ☐ Behavioural & social sciences ☐ Ecological, evolutionary & environmental sciences

For a reference copy of the document with all sections, see nature.com/documents/nr-reporting-summary-flat.pdf

Life sciences study design

All studies must disclose on these points even when the disclosure is negative.

Sample size	All samples available were included with no active exclusion or statistically selected sample size. Sample size was determined based on the availability of pre-existing data, but multiple studies from around the world were combined to provide greater power to all statistical tests.
Data exclusions	Samples were excluded from analysis if they had fewer than 1,000 proteins predicted on them. This avoided inflated misidentification during protein ecology analysis.
Replication	Independent studies are included within the analysis to facilitate replication of results. Analysis of proteins ecology is determinative so provided consistant results.
Randomization	All studies were previously generated and published. No additional randomisation was conducted within this study as patients had been studied prior and no intervention occurred. The combination of multiple studies may enhance the randomisation of patients across conditions.
Blinding	All studies were previously generated and published. Experimentors in this study were not blinded at all.

Reporting for specific materials, systems and methods

We require information from authors about some types of materials, experimental systems and methods used in many studies. Here, indicate whether each material, system or method listed is relevant to your study. If you are not sure if a list item applies to your research, read the appropriate section before selecting a response.

Materials & experimental systems

n/a	Involved in the study
x	Antibodies
x	Eukaryotic cell lines
x	Palaeontology and archaeology
x	Animals and other organisms
x	Clinical data
x	Dual use research of concern
x	Plants

Methods

n/a	Involved in the study
x	ChIP-seq
x	Flow cytometry
x	MRI-based neuroimaging

Plants

Seed stocks	*Report on the source of all seed stocks or other plant material used. If applicable, state the seed stock centre and catalogue number. If plant specimens were collected from the field, describe the collection location, date and sampling procedures.*
Novel plant genotypes	*Describe the methods by which all novel plant genotypes were produced. This includes those generated by transgenic approaches, gene editing, chemical/radiation-based mutagenesis and hybridization. For transgenic lines, describe the transformation method, the number of independent lines analyzed and the generation upon which experiments were performed. For gene-edited lines, describe the editor used, the endogenous sequence targeted for editing, the targeting guide RNA sequence (if applicable) and how the editor was applied.*
Authentication	*Describe any authentication procedures for each seed stock used or novel genotype generated. Describe any experiments used to assess the effect of a mutation and, where applicable, how potential secondary effects (e.g. second site T-DNA insertions, mosiacism, off-target gene editing) were examined.*
